# Sentence-level sentiment analysis based on supervised gradual machine learning

**DOI:** 10.1038/s41598-023-41485-8

**Published:** 2023-09-04

**Authors:** Jing Su, Qun Chen, Yanyan Wang, Lijun Zhang, Wei Pan, Zhanhuai Li

**Affiliations:** School of Computer Science, Northwestern Polytechnical University, Xi’an, 710072 Shaanxi China

**Keywords:** Network models, Software

## Abstract

Sentence-level sentiment analysis (SLSA) aims to identify the overall sentiment polarity conveyed in a given sentence. The state-of-the-art performance of SLSA has been achieved by deep learning models. However, depending on the i.i.d (independent and identically distributed) assumption, the performance of these deep learning models may fall short in real scenarios, where the distributions of training and target data are almost certainly different to some extent. In this paper, we propose a supervised solution based on the non-i.i.d paradigm of gradual machine learning (GML) for SLSA. It begins with some labeled observations, and gradually labels target instances in the order of increasing hardness by iterative knowledge conveyance. It leverages labeled samples for supervised deep feature extraction, and constructs a factor graph based on the extracted features to enable gradual knowledge conveyance. Specifically, it employs a polarity classifier to detect polarity similarity between close neighbors in an embedding space, and a separate binary semantic network to extract implicit polarity relations between arbitrary instances. Our extensive experiments on benchmark datasets show that the proposed approach achieves the state-of-the-art performance on all benchmark datasets. Our work clearly demonstrates that by leveraging DNN for feature extraction, GML can easily outperform the pure DNN solutions.

## Introduction

Sentence-level sentiment analysis (SLSA) aims to analyze the opinions and emotions expressed in a sentence^1^ . Unlike aspect-level sentiment analysis (ALSA)^2^, which reasons about the local sentiment polarity expressed towards a specific aspect, SLSA needs to detect the general sentiment orientation of an entire sentence. In practice, SLSA is highly valuable in the scenarios where comments are represented by concise and isolated sentences with arbitrary topics, requiring a holistic analysis of sentiment at the sentence level. For instance, in e-commerce, platforms (e.g., taobao.com and booking.com) can optimize their marketing strategies by understanding consumers’ preference with the purchasing experience; product manufacturers, such as smartphone or computer producers, can improve product design based on customer feedback. In another application, social media platforms (e.g., Twitter and Facebook) usually analyze people’s comments and posts by SLSA to gain insights into public opinion and social trends.

**Figure 1:**
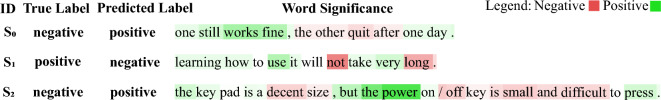
The illustrative examples of SLSA from the CR dataset: the BERT model mispredicts on all the three sentences.

The state-of-the-art performance of SLSA has been achieved by various DNN models. Especially, over the last few years, it has been empirically shown that by semantic learning on large-scale corpus, the pre-trained models (e.g., BERT^3^, RoBERTa^4^ and XLNet^5^) can automatically capture implicit sentimental features, thus effectively improve the performance of SLSA. However, the efficacy of these DNN models depends on the i.i.d (independent and identically distributed) assumption; but in real scenarios, there may not be sufficient labeled training data, and even if provided with sufficient training data, the distributions of training data and target data are almost certainly different to some extent. We illustrate the challenge of SLSA by the running examples as shown in Fig. 1, in which we indicate the sentimental polarities of words by color depths. In $S_0$, the first part expresses a positive polarity, but the polarity of the second part is negative. As a result, its overall polarity is negative. Unfortunately, the BERT model misidentifies its polarity as positive. In $S_1$, the BERT model fails to detect the positive polarity of the combination of “not” and “long”.

To alleviate the limitation resulting from distribution misalignment between training and target data, this paper proposes a supervised approach for SLSA based on the recently proposed non-i.i.d paradigm of Gradual Machine Learning. In general, GML begins with some easy instances, and then gradually labels more challenging instances by knowledge conveyance between labeled and unlabeled instances. Technically, GML fulfills gradual knowledge conveyance by iterative factor inference in a factor graph. It is noteworthy that unlike the i.i.d learning approaches (e.g., deep learning), which train a single unified model for all the instances in a target workload, GML gradually learns about the label status of each instance based on evolving evidential observations. By gradual learning, GML can effectively bridge distribution alignment between labeled training data and unlabeled target data. GML has been successfully applied to the task of Aspect-Level Sentiment Analysis (ALSA)^6,7^ as well as entity resolution^8^. Even without leveraging labeled training data, the existing unsupervised GML solutions can achieve competitive performance compared with supervised DNN models. However, the performance of these unsupervised solutions is still constrained by inaccurate and insufficient knowledge conveyance. For instance, the existing GML solution for aspect-level sentiment analysis mainly leverages sentiment lexicons and explicit polarity relations indicated by discourse structures to enable sentimental knowledge conveyance. On one hand, sentiment lexicons may be incomplete and a sentiment word’s actual polarity may vary in different sentence contexts; on the other hand, explicit polarity relations are usually sparse in natural language corpora. Therefore, their efficacy as the medium for sentimental knowledge conveyance is limited.

**Figure 2:**
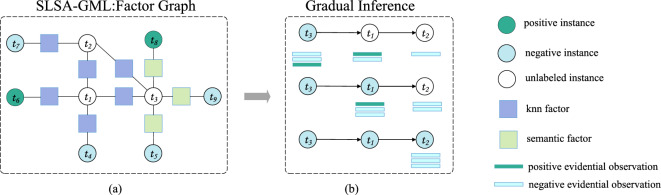
An illustrative example of gradual learning: 1) subgraph (a) represents a factor graph that connects unlabeled and labeled samples with two types of relational features (knn features and semantic features); 2) subgraph (b) illustrates gradual inference process on unlabeled samples based on their evidential observations. The labels of $t_3$, $t_1$ and $t_2$ are subsequently inferred to be negative.

Our proposed GML solution for SLSA aims to effectively exploit labeled training data to enhance gradual learning. Specifically, it leverages binary polarity relations, which are the most direct way of knowledge conveyance, to enable supervised gradual learning. Since it has been widely recognized that BERT-based pre-trained models can capture sentimental features more accurately than manually crafted features (e.g., sentiment lexicons), we leverage labeled training data to extract sentiment features by BERT-based models. Similar to the existing DNN models, it trains a sentence-level polarity classifier such that the sentences with similar polarities can be clustered within local neighborhood in a deep embedding space. To enable knowledge conveyance beyond local neighborhood, we also separately train a semantic network to extract implicit polarity relations between two arbitrary sentences. All the extracted features are then modeled as binary factors in a factor graph to fulfill gradual learning. We have illustrated the process of gradual inference by the example in Fig. 2. Gradual knowledge conveyance is supposed to be enabled by binary factors. In the example, given the evidential observations and the binary similarity factors, the labels of $t_3$, $t_1$ and $t_2$ can be subsequently reasoned to be negative.

The major contributions of this paper can be summarized as follows:

- We present a supervised GML solution for SLSA, which can effectively exploit labeled training data to enhance gradual learning;
- We present two types of DNN models to capture implicit sentimental features, and model them as binary factors in a factor graph to fulfill supervised knowledge conveyance for SLSA;
- We empirically validate the efficacy of the proposed solution on real benchmark workloads by a comparative study. Our extensive experiments have shown that it consistently achieves the state-of-the-art performance across all the test workloads.

The rest of this paper is organized as follows. Section “Related work” discusses related work. “Preliminaries” defines the task of SLSA and introduces the GML framework. “Supervised GML solution” presents the proposed solution. “Empirical evaluation” empirically evaluates the proposed solution. In “Conclusion”, we conclude this paper with some thoughts on future work.

## Related work

Sentiment analysis has been extensively studied at different granularities (e.g., document-level, sentence-level and aspect-level) in the literature. At the document level, the goal is to detect the sentiment polarity of an entire review, which may be composed of multiple sentences. Sentence-level sentiment analysis aims to detect the general polarity expressed in a single sentence. Representing the finest granularity, aspect-level sentiment analysis needs to identify the polarity expressed towards certain aspects of entity within a sentence. It is noteworthy that a sentence may express conflicting polarities towards difference aspects in a sentence. The state-of-the-art solutions for sentiment analysis at different granularities have been built upon DNN models. For instance, for document-level sentiment analysis, the DNN models include CSNN^9^, AttBiLSTM-2DCNN^10^, CNN-BiLSTM^11^, SR-LSTM^12^ and BAE^13^; for aspect-level sentiment analysis, the most recent DNN models include LCF-BERT^14^, PTMs^15^ and RGAT^16^, all of which are variants of the pre-trained BERT model. This paper focuses on sentiment analysis at sentence level. In the rest of this section, we review related work from the orthogonal perspectives of sentence-level sentiment analysis and gradual machine learning.

### Sentence-level sentiment analysis

Early work on SLSA mainly focused on extracting different sentiment hints (e.g., n-gram, lexicon, pos and handcrafted rules) for SVM classifiers^17–20^. Unfortunately, these features are either sparse, covering only a few sentences, or not highly accurate. The advance of deep neural networks made feature engineering unnecessary for many natural language processing tasks, notably including sentiment analysis^21–23^. More recently, various attention-based neural networks have been proposed to capture fine-grained sentiment features more accurately^24–26^. Unfortunately, these models are not sufficiently deep, and thus have only limited efficacy for polarity detection.

Most recently, the research on SLSA has experienced a considerable shift towards large pre-trained Language models (e.g., BERT, RoBERTa and XLNet)^4,5,27,28^. Some researchers investigated how to integrate the traditional language features (e.g., part-of-speech, syntax dependency tree and knowledge-base) into pre-trained models for improved performance^27,29,30^. Other researchers focused on how to design new networks for sentiment analysis based on the standard transformer structure^28,31^. Typically, they fed the outputs of the BERT model to a new network, reloading the parameters of the original pre-trained model to a new network. Subsequently, several new pre-training proposals have been presented to mitigate the mismatch between a new network structure and a pre-trained model^27,28^. For instance, SentiLARE encoded sentiment score as part of input embedding and performed post-pretraining on the yelp datasets to get its own pre-trained model^27^. The work of Entailment modified the pre-training process to generate a new pre-trained model SKEP_ERNIE_2.0_LARGE_EN^28^ .

Furthermore, to better adapt a pre-trained model to downstream tasks, some researchers proposed to design new pre-training tasks^28,32^. For instance, the work of SentiBERT designed specific pre-training tasks to guide a model to predict phrase-level sentiment label^32^. The work of Entailment reformulated multiple NLP tasks, which include sentence-level sentiment analysis, into a unified textual entailment task^28^. It is noteworthy that so far, this approach achieved the state-of-the-art performance on sentence-level sentiment analysis.

It is noteworthy that all the above-mentioned deep learning solutions for SLSA were built upon the i.i.d learning paradigm. For a down-stream task of SLSA, their practical efficacy usually depends on sufficiently large quantities of labeled training data. However, in real scenarios, there may not be sufficient labeled training data, and even if provided with sufficient training data, the distributions of training data and target data are almost certainly different to some extent.

### Gradual machine learning

The non-i.i.d learning paradigm of gradual machine learning (GML) was originally proposed for the task of entity resolution^8^. It can gradually label instances in the order of increasing hardness without the requirement for manual labeling effort. Since then, GML has been also applied to the task of aspect-level sentiment analysis^6,7^. It is worthy to point out that as a general paradigm, GML is potentially applicable to various classification tasks, including sentence-level sentiment analysis as shown in this paper. Even though the existing unsupervised GML solutions can achieve competitive performance compared with many supervised approaches, without exploiting labeled training data, their performance is still limited by inaccurate and insufficient knowledge conveyance. In this paper, we focus on how to supervise feature extraction by DNNs and leverage them for improved gradual learning on the task of SLSA.

## Preliminaries

In this section, we first define the SLSA task, and then provide a brief overview of the GML framework.

### Task definition

As usual, this paper considers SLSA as a binary classification problem, in which a classifier needs to label each sentence as *positive* or *negative*. Formally, we define the task of SLSA as follows:

#### Definition 1

(*Sentence-level sentiment analysis*) Given a corpus of reviews{$r_0$,$r_1$,$r_2$,$\ldots$,$r_n$ }, each review $r_j$ consisting of a sequence of sentences, {$s_{j1}$,$s_{j2}$,$\ldots$,$s_{jm}$}, the goal of SLSA is to predict the label of each sentence, where the label can be either positive ($label = 1$) or negative ($label = 0$).

### The general GML framework

**Figure 3:**
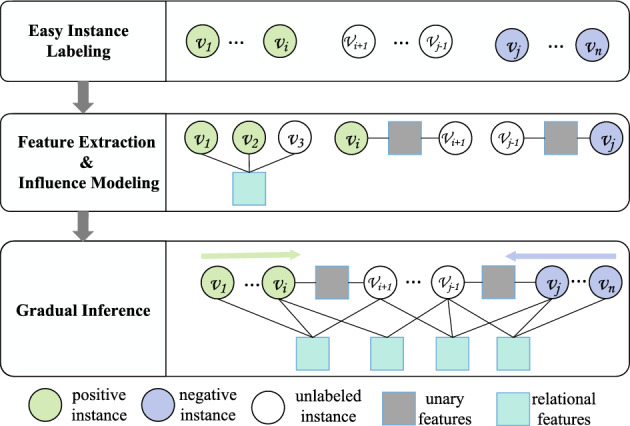
The general GML framework.

As shown in Fig. 3, the general GML framework consists of the following three essential steps:

#### Easy instance labeling

Gradual machine learning begins with the label observations of easy instances. In the unsupervised setting, easy instance labeling can usually be performed based on the expert-specified rules or unsupervised learning. For instance, it can be observed that an instance usually has only a remote chance to be misclassified if it is very close to a cluster center. Therefore, it can be considered as an easy instance and automatically labeled.

For aspect-level sentiment analysis, it has been shown^6^ that if a sentence contains some strong positive (res. negative) sentiment words, but no negation, contrast and hypothetical connectives, it can be reliably reasoned to be positive (res. negative). In this paper, we study sentence-level sentiment analysis in the supervised setting, in which some labeled training data are supposed to be available. These training instances with ground-truth labels can naturally serve as initial easy instances.

#### Feature extraction and influence modeling

In GML, features serve as the medium for knowledge conveyance between labeled and unlabeled instances. A wide variety of features usually need to be extracted to capture diverse information. For each type of feature, this step also needs to model its influence over label status. It is common that different applications require different features. In our previous work on unsupervised GML for aspect-level sentiment analysis^6^, we extracted sentiment words and explicit polarity relations indicated by discourse structures to facilitate knowledge conveyance. Unfortunately, for sentence-level sentiment analysis, polarity relation hints seldom exist between sentences, and sentiment words are usually incomplete and inaccurate. Therefore, we propose to use DNNs to extract implicit sentiment features.

#### Gradual inference

This step gradually labels the instances with increasing hardness in a workload. GML fulfills gradual learning by iterative factor inference over a factor graph consisting of the labeled and unlabeled instances and their common features. At each iteration, it typically labels the unlabeled instance with the highest degree of evidential certainty.
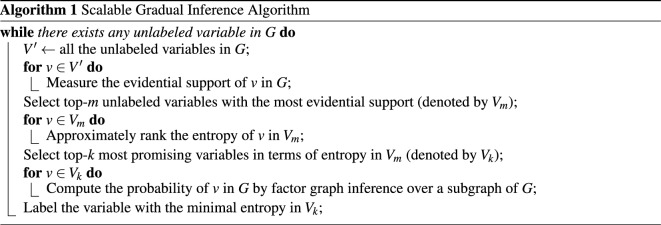


Formally, suppose that the total number of class labels, denoted by {$L_1,L_2,\ldots ,L_t$}, is *t*. Given an inference variable *v*, GML measures its evidential certainty by the inverse of entropy as follows

1 $$\begin{aligned} E(v)=\frac{1}{H(v)} =\frac{1}{- \sum \nolimits _{1\le i\le t}{P_i({v})} \cdot {\log _2}P_i({v})}, \end{aligned}$$

in which *E*(*v*) and *H*(*v*) denote the evidential certainty and entropy of *v* respectively, and $P_i(v)$ denotes the inferred probability of *v* having the label of $L_i$. It is noteworthy that in the process of gradual inference, a newly labeled instance at the current iteration would serve as an evidence observation in the following iterations.

In practice, GML is usually implemented by scalable gradual inference, which was first proposed for the task of entity resolution^8^. We have sketched the general process of scalable gradual inference, which is the same as presented in^8^, in Algorithm 1. It consists of the following three steps: (1) measurement of evidential support; (2) approximate ranking of entropy; (3) factor subgraph inference. Given a factor graph *G*, it first selects the top-*m* unlabeled variables with the most evidential support in *G* as the candidates for probability inference. To reduce the invocation frequency of factor graph inference, it then approximates entropy estimation by an efficient algorithm on the *m* candidates and selects only the top-*k* most promising variables among them for factor graph inference. Finally, it infers the probabilities of the chosen *k* variables by factor subgraph inference.

## Supervised GML solution

The supervised solution directly uses labeled examples in training data as easy instances. As mentioned in the introduction, it leverages two types of DNN models for knowledge conveyance: a polarity classifier, which is supposed to extract polarity-sensitive vector representations for detecting polarity similarity between close neighbors in a deep embedding space, and a semantic deep network, which can detect both similar and opposite polarity relations between two arbitrary sentences. Then, our solution constructs a factor graph consisting of both unary and binary factors to enable gradual learning.

**Figure 4:**
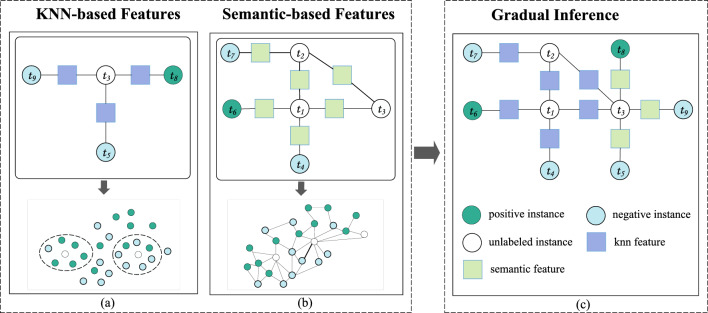
The illustration of supervised GML solution for SLSA: it extracts two types of polarity relation features, one based on local neighborhood and the other based on semantic deep network.

We illustrate the proposed solution by the example as shown in Fig. 4. In the example, there are three unlabeled instances: $t_1$, $t_2$ and $t_3$. The subfigures (a) and (b) show their neighborhood-based similarity features and semantic relation features respectively, and the subfigure (c) shows the constructed factor graph. In the rest of this section, we first present the DNN models to extract polarity relation features, and then describe how to model them as factors in a factor graph to facilitate gradual learning. For presentation simplicity, we have summarized the frequently used notations in Table 1.

**Table 1:** Frequently used notations.

Notation	Description
*v*	Affective vector
*h*	Hidden states vector
$h_{a}$	Affective-aware hidden states vector
*w*	Affective attention weight vector
$w^i$	Affective attention weight of a word i
$w_d$	The *d*-th dimensional affective weight (e.g., the weight of Evaluation)
$v_d^i$	The *d*-th dimension affective value of a word i (e.g., the sentiment score of word i in evaluation)

### Feature extraction: polarity relations

#### Similarity relations by polarity classifier

Since recent work has shown that encoding handcrafted affective knowledge (e.g., sentiment lexicons) can effectively enhance the training of deep DNNs for polarity classification^33^, our solution adds a new branch of sentiment attention upon the EFL-based DNN model^28^ to generate a polarity-sensitive embedding space.

**Figure 5:**
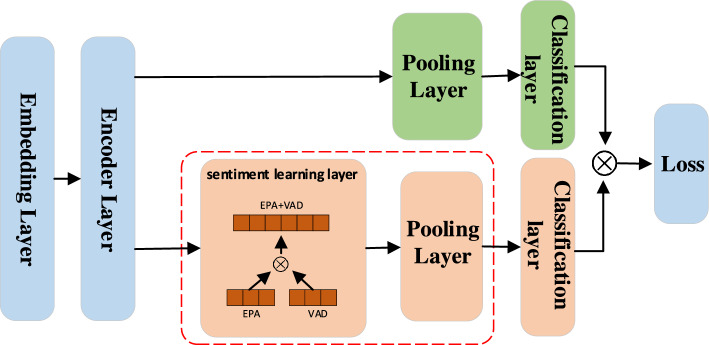
The structure of EFL-based polarity classification model: 1) the boxes circled in red dashed lines represent the newly added branch of sentiment attention encoding affective knowledge; 2) the orange blocks represent the components of the newly added sentiment learning layer, the green blocks are the ones present in the original EFL model, and the blue blocks are the shared components between these two branches.

The new branch of sentiment attention, which consists of a sentiment learning layer and a pooling layer, serves to reflect the explicit sentiment polarities of each word as indicated by sentiment lexicons. In the sentiment learning layer, we build affective vectors by using two sentiment lexicons, EPA (evaluation, potency, and activity) and VAD (valence, arousal, and dominance), both of which measure sentiment orientation by three separate dimensions of continuous numerical values. Specifically, we concatenate the two word-level vectors of each word into a 6-dimensional affective vector, *v*, where the six dimensions correspond to evaluation, potency, activity, valence, arousal, and dominance, respectively. In the model, the output of the encoder layer is a vector of hidden states denoted by $h\in {\mathscr {R}}^{b\times m \times e}$, which is then fed to the pooling layer, in which *b* denotes the batch size, *m* denotes the max length of sequence, *e* denotes the embedding dimension. Before conveying *h* to the pooling layer, we transform *h* into a new vector of affective-aware hidden states, which is denoted by $h_{a}$. Specifically, we measure the attention weight of each sentiment word by the weighted sum of its sentiment dimension values as follows:

2 $$\begin{aligned} w^i = 1 + \sum _{d=0}^{5}(w_d \times v_d^i), \end{aligned}$$

in which $w^i$ denotes the attention weight of a sentiment word, $w_d$ denotes the *d*-th affective dimension weight, and $v_d^i$ denotes the word’s sentiment value in the *d*-th affective dimension. Note that the values of $w_d$ represents the weights of six dimensions (namely evaluation, potency, activity, valence, arousal and dominance); in our implementation, we set their values at [0.2, 0.2, 0.3, 0.3, 0.2, 0.2] as suggested by^33^. The value of $v_d^i$ denotes a word’s sentiment value in the *d*-th affective dimension, which can be directly extracted from the EPA and VAD lexicons. It is also noteworthy that the dimension value domain of the EPA lexicon is [0,1], while the dimension value domain of the VAD is [− 5,5]. Therefore, we use a mapping function to unify the domains of EPA and VAD at [0,1]. Based on the setting of $w_d$ and $v_d^i$, the value domain of $w^i$ is between 1 and 2.4. If a word is absent in the sentiment lexicons, we set its attention weight as 1, or $w^i=1$, effectively ignoring the lexicon influence.

Next, we concatenate the attention weights of all the words in a sentence to obtain its affective attention weight vector *w* as follows:

3 $$\begin{aligned} w = \left[ w^1,w^2,\ldots ,w^m\right] \end{aligned}$$

where $w\in {\mathscr {R}}^{m\times 1}$. Then, we compute the new affective-aware hidden states vector $h_{a}$ by weighting the original hidden states vector *h* with *w* as follows:

4 $$\begin{aligned} h_{a} = w \times h. \end{aligned}$$

In the final classification layer, we fuse the features from both branches as follows:

5 $$\begin{aligned} l^{'} = l_{\eta } + \theta \times l_{\xi }, \end{aligned}$$

in which $l_{\eta }$ and $l_{\xi }$ denote the losses generated by the representation learning branch and the sentiment attention branch respectively, and $\theta$ denotes the penalty weight parameter to balance the contributions from two branches. Since the sentiment attention branch is supposed to complement the main representation learning branch, we suggest to set the value of $\theta$ at less than 0.5. In our implementation, we set $\theta = 0.3$.

Specifically, we have

6 $$\begin{aligned} l_{\eta } =y_{\eta }\log {\hat{y}}+(1-y_{\eta })\log (1-{\hat{y}}), \end{aligned}$$

and

7 $$\begin{aligned} l_{\xi } =y_{\xi }\log \hat{y}+(1-y_{\xi })\log (1-\hat{y}). \end{aligned}$$

We fine-tune the polarity classification model as shown in Fig. 5 using labeled training data, and then exploit the resulting vector representations (the last-layer embeddings) for polarity similarity detection. In the implementation, we have constructed the DNN of polarity classification based on the state-of-the-art EFL model^28^. For each unlabeled sentence in a target workload, we extract its *k*-nearest neighbors from both the labeled and unlabeled instances. We use cosine distance to measure similarity. The value of *k* is usually set to a small number to ensure the accuracy of extracted relations. Furthermore, we use a threshold (e.g., 0.001 in our experiments) to filter out the nearest neighbors not close enough in the embedding space. Our experiments have demonstrated that the performance of supervised GML is robust w.r.t the value of *k* provided that it is set within a reasonable range (between 1 and 9).

#### Similarity/opposite relations by semantic deep network

**Figure 6:**
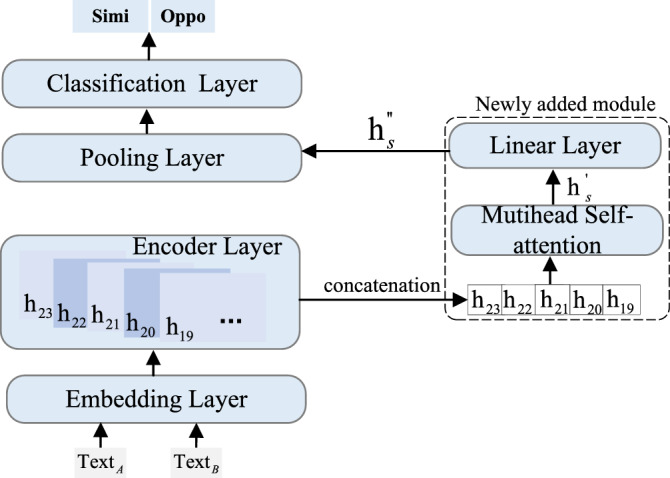
The structure of semantic deep network for arbitrary polarity relation extraction: the dotted box on the right side denotes our newly added module.

Built upon the transformer architecture, the semantic deep network aims to detect the polarity relation between two arbitrary sentences. The backbone of a transformer is an encoder consisting of multiple multi-head self-attention layers. Each layer has the same network structure but different parameter weights. It has been well recognized that in a transformer, besides the last hidden layer, other layers also contain sentimental information^34^. Therefore, we add a self-attention layer to aggregate the information present in the last five layers of a transformer, and use a super feature vector to capture additional sentimental features beyond the last layer.

Specifically, as shown in Fig. 6, the structure of the semantic deep network can be represented by the following three equations:

8 $$\begin{aligned}{} & {} h_{s} =h_{19}\oplus h_{20}\oplus h_{21}\oplus h_{22}\oplus h_{23}, \end{aligned}$$

9 $$\begin{aligned}{} & {} {h_{s}}^{'} =f_{m}(h_{s}), \end{aligned}$$

10 $$\begin{aligned}{} & {} {h_{s}}^{''} =w_{s} \times {h_{s}}^{'} + b_{s}. \end{aligned}$$

In Eq. (8), $\oplus$ denotes the concatenation operation, $h_{i}$ represents the vector output of the previous ith hidden layer, $i\in${19,20,21,22,23} and $h_{i}\in {\mathscr {R}}^{b\times m \times e}$, in which *b* denotes the batch size, *m* denotes the max length of sequence, *e* denotes the embedding dimension, ${h_{s}}$ denotes the super vector after concatenation, ${h_{s}}\in {\mathscr {R}}^{ b\times m \times 5e}$. Equation (9) exploits the muti-head self attention function $f_m$ on the super vector to learn more different features. Finally, Eq. (10) employs a linear function to map dimension size of $b \times m \times 5e$ to the original dimension size of $b \times m \times e$ for subsequent layers, where $w_{s} \in R^{5e\times e}$ denotes a weight matrix and $b_s$ denotes the bias, both of which are supposed to be learned by training .

Provided with the super vector of $h_{s}$, the first step of the multi-head self-attention function is to map the raw input vector to a query vector, a key vector and a value vector by linear transformation as follows:

11 $$\begin{aligned} q_s= & {} w_q\times h_{s}+ b_q, \end{aligned}$$

12 $$\begin{aligned} k_s= & {} w_k \times h_{s}+b_k, \end{aligned}$$

13 $$\begin{aligned} v_s= & {} w_v \times h_{s}+ b_v, \end{aligned}$$

in which $q_s$, $k_s$ and $v_s$ denote query vector, key vector and value vector respectively, $w_q$, $w_k$ and $w_v$ denote weight matrix with the size of $R^{b\times m\times 5 e}$, and $b_q$, $b_k$, $b_v$ denote bias.

Then, it exploits a softmax function to convert the query vector and key vector into an attention probability as follows:

14 $$\begin{aligned} a_p = Softmax\left( \frac{q_s \times {k_s}^T}{\sqrt{\gamma }} \right) \end{aligned}$$

in which $a_p\in {\mathscr {R}}^{b\times m \times m}$. Finally, it multiplies $a_p$ with $v_s$ to get a combined context-informed word feature ${h_{s}}^{'}$ as follows:

15 $$\begin{aligned} {h_{s}}^{'} = a_p\times v_s \end{aligned}$$

Then, it transforms ${h_{s}}^{'}$ to ${h_{s}}^{''}$ according to Eq. (10). It subsequent processing is similar to the traditional transformer architecture.

For SLSA, we construct polarity relations between labeled and unlabeled sentences based on a trained semantic deep network. In the training phase, we randomly extract *r* labeled sentences from training data for each labeled sentence to fine-tune the semantic network. Then, in the feature extraction phase, we randomly extract *r* sentences from labeled training data for each unlabeled sentence in the target workload, and construct its relations w.r.t them based on the semantic network. Our experiments has demonstrated that the performance of supervised GML is very robust w.r.t the value of *r* provided that it is set within a reasonable range ($3\le r\le 8$).

### Factor modeling of binary relations

A factor graph for gradual machine learning consists of evidential variables, inference variables and factors. In the case of SLSA, a variable corresponds to a sentence and a factor defines a binary relation between two variables. In the process of GML, the labels of inference variables need to be gradually inferred. The label of a variable once inferred remains unchanged. The factor graph of the illustrative example has been shown in Fig. 4.

In the supervised setting, all the labeled training data serve as the easy instances. The labels of the sentences in a target workload need to be gradually inferred by knowledge conveyance through the extracted binary relations. Specifically, we define the binary factor of a KNN-based similarity relation, $f_k$, as

16 $$\begin{aligned} \varphi _{f_k}(v_i, v_j) = \left\{ \begin{array}{ll} e^{w_{f_k}} &{} if \ v_i = v_j; \\ 1 &{} otherwise; \end{array} \right. \end{aligned}$$

where $v_i$ and $v_j$ denote the two variables sharing the KNN-based similarity relational feature $f_k$, and $w_{f_k}$ denotes the weight of $f_k$. Similarly, we define the binary factor of a semantic relation between two variables, $f_s$, as

17 $$\begin{aligned} \varphi _{f_s}(v_i, v_j) = \left\{ \begin{array}{ll} e^{w_{f_s}} &{} if \ v_i = v_j; \\ 1 &{} otherwise; \end{array} \right. \end{aligned}$$

where $v_i$ and $v_j$ denote the two variables sharing the semantic relational feature $f_s$, and $w_{f_s}$ denotes the weight of $f_s$.

In our implementation of scalable gradual inference, the same type of factors are supposed to have the same weight. Initially, the weights of the similarity factors (whether KNN-based or semantic factors) are set to be positive (e.g., 1 in our experiments) while the weights of the opposite semantic factors are set to be negative (e.g., − 1 in our experiments). It is noteworthy that the weights of three parameters would be continuously learned based on evidential observations in the inference process.

On the computational complexity of scalable gradual inference, the analytical results on SLSA are essentially the same as the results represented in our previous work on ALSA^6^. Specifically, in each labeling iteration, the computational complexity of evidential support measurement can be represented by $O(n\times n_f)$, in which n denotes the total number of instances in a workload, and $n_f$ denotes the total number of extracted features; the computational complexity of approximate entropy estimation can be represented by $O(m\times n_f)$, in which *m* denotes the number of candidate variables selected for approximate entropy estimation as specified in Algorithm 1; the computational complexity of factor subgraph construction can be represented by $O(k\times n_f)$, in which *k* denotes the number of candidate variables selected for factor graph inference as specified in Algorithm 1.

## Empirical evaluation

In this section, we empirically evaluate the performance of the proposed solution by a comparative study. “Experimental setup” describes the experimental setup. “Comparative evaluation”* presents the comparative evaluation results. “Sensitivity evaluation” evaluates performance sensitivity of the proposed solution w.r.t algorithmic parameters. “Discussion on extendability to other classification tasks”* discusses the proposed approach’s extendability to other classification tasks.

**Table 2:** The statistics of the test datasets.

Dataset	Train	Validation	Test
MR	8534	1066	1050
CR	2262	754	754
Twitter2013	5098	915	2034
SST	6920	872	1821

### Experimental setup

For comparative evaluation, we use the benchmark datasets of movie review (MR), customer review (CR), Twitter2013 and Stanford Sentiment Treebank (SST). Both MR and SST are movie review collections, CR contains the customer reviews of electronic products, while Twitter2013 contains microblog comments, which are usually shorter than movie and product reviews. The detailed statistics of the test datasets are presented in Table 2.

**Table 3:** Comparative evaluation results: we highlight the best results on each dataset in bold.

Model	CR		MR		SST		Twitter2013	
	Acc	Macro-F1	Acc	Macro-F1	Acc	Macro-F1	Acc	Macro-F1
EFL	93.94% $\pm {0.04}$	95.36% $\pm {0.12}$	92.27% $\pm {0.32}$	92.23% $\pm {0.22}$	94.51% $\pm {0.02}$	94.60% $\pm {0.40}$	93.36% $\pm {0.55}$	95.38% $\pm {0.45}$
SentiLARE	92.41% $\pm {0.14}$	94.03% $\pm {0.11}$	91.52% $\pm {0.10}$	91.42% $\pm {0.15}$	94.56% $\pm {0.80}$	94.69% $\pm {0.23}$	92.90% $\pm {0.26}$	95.02% $\pm {0.24}$
RoBERTa-Large	93.73% $\pm {0.43}$	95.06% $\pm {0.27}$	92.13% $\pm {0.25}$	92.12% $\pm {0.28}$	95.66% $\pm {0.91}$	95.75% $\pm {0.64}$	94.36% $\pm {0.44}$	96.10% $\pm {0.30}$
XLNET-Large	93.44% $\pm {0.07}$	94.83% $\pm {0.04}$	91.31% $\pm {0.26}$	91.33% $\pm {0.25}$	95.30% $\pm {0.19}$	95.40% $\pm {0.73}$	93.80% $\pm {0.55}$	95.72% $\pm {0.50}$
RoBERTa-Base	93.04% $\pm {0.46}$	94.50% $\pm {0.31}$	90.23% $\pm {0.23}$	90.27% $\pm {0.21}$	94.82% $\pm {0.19}$	95.00% $\pm {0.13}$	93.51% $\pm {0.37}$	95.50% $\pm {0.31}$
XLNET-Base	92.84% $\pm {0.28}$	94.40% $\pm {0.15}$	90.09% $\pm {0.29}$	90.16% $\pm {0.73}$	93.00% $\pm {0.38}$	93.17% $\pm {0.38}$	92.51% $\pm {0.09}$	94.84% $\pm {0.09}$
SBERT	92.93 % $\pm {0.10}$	93.55 % $\pm {0.32}$	92.38% $\pm {0.45}$	92.58% $\pm {0.40}$	95.09% $\pm {0.80}$	95.22% $\pm {0.21}$	93.25% $\pm {0.11}$	94.31% $\pm {0.32}$
AGN	91.89% $\pm {0.13}$	91.24% $\pm {0.20}$	87.60% $\pm {0.32}$	87.57% $\pm {0.13}$	92.72% $\pm {0.20}$	90.94% $\pm {0.29}$	91.26% $\pm {0.30}$	91.26% $\pm {0.45}$
DualCL	92.19% $\pm {0.28}$	92.68% $\pm {0.85}$	89.41% $\pm {0.54}$	89.06% $\pm {0.13}$	93.41% $\pm {0.10}$	93.58% $\pm {0.77}$	88.94% $\pm {0.24}$	89.05% $\pm {0.22}$
SLSA-GML	**95.62**% $\pm {0.21}$	**96.54**% $\pm {0.29}$	**93.16**% $\pm {0.30}$	**93.04**%$\pm {0.32}$	**96.27**% $\pm {0.12}$	**96.30**% $\pm {0.15}$	**94.94**% $\pm {0.20}$	**96.49**% $\pm {0.20}$

**Table 4:** The evaluation results of ablation study: we separately evaluate the efficacy of the KNN and semantic factors.

Model	CR		5MR		SST		Twitter2013	
	Acc	Macro-F1	Acc	Macro-F1	Acc	Macro-F1	Acc	Macro-F1
SLSA-GML (w/o knn)	95.36% $\pm {0.20}$	96.32% $\pm {0.35}$	93.07% $\pm {0.17}$	92.97% $\pm {0.72}$	96.16% $\pm {0.71}$	96.20% $\pm {0.45}$	94.74% $\pm {0.23}$	96.34% $\pm {0.85}$
SLSA-GML (w/o semantic)	94.56% $\pm {0.75}$	95.69% $\pm {0.25}$	93.06% $\pm {0.89}$	92.94% $\pm {0.65}$	95.61% $\pm {0.27}$	95.66% $\pm {0.55}$	93.90% $\pm {0.86}$	95.78% $\pm {0.95}$
SLSA-GML	**95.62**% $\pm {0.74}$	**96.54**% $\pm {0.06}$	**93.16**% $\pm {0.28}$	**93.04**% $\pm {0.90}$	**96.27**% $\pm {0.39}$	**96.30**% $\pm {0.43}$	**94.94**% $\pm {0.41}$	**96.49**% $\pm {0.11}$

Significant values are in [bold].

Because the recent proposed DNN solutions based on pre-trained language models have been empirically shown to outperform earlier proposals, we compare the proposed solution, denoted by GML, with the following state-of-the-art models:

- EFL^28^. Converting class labels into auxiliary sentences, it is a unified model that can model multiple NLP tasks as a textual entailment task.
- SentiLARE^27^. As a language representation model, it introduces word-level linguistic knowledge, which include part-of-speech tag and sentiment polarity, into pre-trained models and uses a label-aware masked language model to construct knowledge-aware language representation.
- RoBERTa-Large^4^. It purposedly removes the next sentence prediction objective and dynamically changes masking pattern to improve the performance on downstream tasks.
- XLNet-Large^5^. It is based on a generalized autoregressive pre-trained model that can learn bidirectional contexts by maximizing the expected likelihood over all the permutations of factorization order.
- RoBERTa-Base^4^. It is a simpler version of RoBERTa-Large with only 12 hidden layers.
- XLNet-Base^5^. It is a simpler version of XLNet-Large with only 12 hidden layers.
- SBERT^32^. It use the siamese and triplet network structures to derive semantically meaningful sentence embeddings for sentimental polarity detection.
- AGN^35^. It integrates statistical information with semantic representation to train a robust classifier for sentiment analysis.
- DualCL^36^. It is a recently proposed framework for sentiment analysis that can simultaneously learn the features of input samples and the parameters of classifiers in the same embedding space.

We have implemented the proposed GML solution based on the open-sourced GML inference engine (https://github.com/gml-explore/gml). Our implementation uses the affective-aware EFL model^28^ as the baseline polarity classifier and leveraged external affective knowledge to extract the KNN-based similarity relations. It uses the improved RoBERTa-Large model^4^ to extract similar and opposite semantic relations between two arbitrary sentences. Specifically, the RoBERTa-Large model consists of 16 heads and 24 layers with the hidden layer size of 1024. Our implementation keeps the dropout probability at 0.1 and sets the number of epochs to 3. It set the initial learning rate at $2e^{-5}$ for all the layers and the batch size at 32. For the training of semantic deep network, we generate 6 semantic relations (3 with similar labels and 3 with opposite labels) for each labeled example. For GML factor graph construction, we randomly select 6 labeled examples in the training set for each unlabeled sentence, and use the trained binary semantic model to predict their polarity relations.

As usual, we measure the performance of different solutions by the metrics of Accuracy and Macro-F1. All the reported results are averages over 5 runs. We report both the means and standard deviations (STD). All the comparative experiments have been conducted on the same machine, which runs the Ubuntu 16.04 operating system and has a NVIDIA GeForce RTX 3090 GPU, 128 GB of memory and 2 TB of solid-state drive.

### Comparative evaluation

The detailed evaluation results have been presented in Table 3. It can be observed that GML consistently achieves the state-of-the-art performance across all the test workloads in terms of Accuracy and Macro-F1. Specifically, in terms of accuracy, GML outperforms the existing best performer, EFL, by the margins of 1.68%, 0.89%, 1.76%, 1.58% on CR, MR, SST and Twitter2013 respectively. Similarly, in terms of macro-F1, GML outperforms EFL by the margins of 1.18%, 0.81%, 1.7%, 1.11% on them respectively. In terms of accuracy, GML outperforms the existing state-of-the-art by around 1.6% on CR, 0.7% on MR, 0.6% on SST, and 0.58% on Twitter2013. In terms of Macro-F1, the improvement margins over the state-of-the-art on the four test workloads are 1.18%, 0.46%, 0.55%,0.40% respectively. It is noteworthy that the performance of two recent approaches, AGN and DualCL, is similar to other DNN models, but worse than GML. We have observed that the statistical features (e.g., word frequency), which are the focus of AGN, are not very helpful to sentiment analysis. As an augmentation approach, DualCL usually performs well in the circumstance where there are only a few labeled training data. In our scenarios of benchmark workloads, the efficacy of DualCL is however rather limited. By leveraging the state-of-the-art DNNs for feature extraction, non-i.i.d gradual learning has a clear advantage over i.i.d learning. It can also be observed that for both GML and deep learning models, the fluctuations of different runs remain low (STD values < 0.5 in most cases). Due to the well recognized challenge of SLSA, these observations clearly indicate the efficacy of the proposed approach.

#### Ablation study

We have also conducted an ablation study on the proposed GML solution. The detailed evaluation results have been reported in Table 4. It can be observed that without either KNN-based relations or binary semantic relations, the performance of GML drops on all the test workloads. This observation clearly indicates that KNN-based relations and binary semantic relations are complementary to each other: their combined modeling in GML achieves better performance than either of them. However, it can also be observed that compared with knn relations, the performance of GML drops more considerably without binary semantic relations. The knn relations capture only similarity features, while the binary semantic relations can capture both similarity and opposite, or more diverse, relations. It is noteworthy that our experimental results are consistent with the expected characteristic of GML that more diverse features can usually facilitate knowledge conveyance more effectively.

#### Illustrative examples

**Figure 7:**
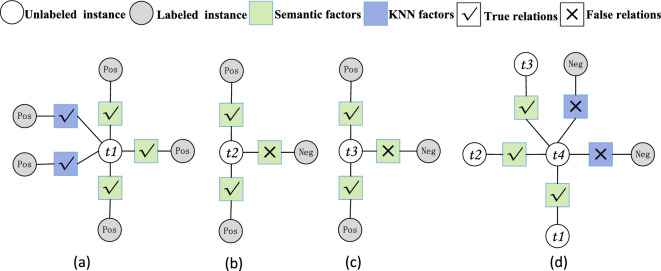
An illustrative example of gradual inference: 1) the four subfigures denote the constructed factor subgraphs of the four examples in Table 5 respectively; 2) a true relation factor (resp. false relation factor) means that its corresponding polarity relation is true (resp. false).

We illustrate the efficacy of GML by the examples from CR as shown in Table 5 and Figure 7. On $t_1$, both GML and the deep learning model give the correct label; however, on all the other examples, GML gives the correct labels while the deep learning model mispredicts. In Figure 7, the four subfigures show the constructed factor subgraphs of the examples respectively. It can be observed that $t_2$ has three relational factors, two of which are correctly predicted while the remaining one is mispredicted. However, GML still correctly predicts the label of $t_2$ because the majority of its relational counterparts indicate a positive polarity. The similar result has also been observed on $t_3$. It is noteworthy that GML labels these examples in the order of $t_1$, $t_2$, $t_3$ and $t_4$. Since the predicted labels of $t_2$ and $t_3$ provide $t_4$ labeling with correct polarity hints, $t_4$ is also correctly labeled as positive.

**Table 5:** The illustrative examples for GML efficacy by the examples from the CR dataset; the column of GML represents the prediction results of our proposed GML solution, while the column of DNN represents the prediction results of the current SOTA DNN model.

#Id	#Text	Ground_Truth_Label	GML	DNN
$t_{1}$	This camera is perfect for an enthusiastic amateur photographer .	Pos	Pos	Pos
$t_{2}$	But i ’ve already emailed creative tech support about it , and gotten timely responses - they will fix it for me if necessary.	Pos	Pos	Neg
$t_{3}$	This did manage to cut the number of pop ups during start-up from 4 down to 2.	Pos	Pos	Neg
$t_{4}$	i imagine if i left my player untouched ( no backlight ) it could play for considerably more than 12 hours at a low volume level.	Pos	Pos	Neg

**Table 6:** The evaluation results of parameter sensitivity: the performance sensitivity of GML w.r.t the number of extracted semantic relations $k_s$ and the number of extracted KNN relations $k_n$.

$k_{n}$ $k_{s}$	CR		MR		SST		Twitter2013	
	Acc	Macro-F1	Acc	Macro-F1	Acc	Macro-F1	Acc	Macro-F1
3 3	95.09% $\pm {0.13}$	96.13% $\pm {0.21}$	92.88% $\pm {0.20}$	92.76% $\pm {0.11}$	96.21% $\pm {0.21}$	96.24% $\pm {0.19}$	95.03% $\pm {0.47}$	96.56% $\pm {0.29}$
3 5	95.76% $\pm {0.23}$	96.64% $\pm {0.27}$	93.35% $\pm {0.17}$	93.22% $\pm {0.17}$	96.32% $\pm {0.20}$	96.36% $\pm {0.22}$	94.54% $\pm {0.43}$	96.21% $\pm {0.27}$
3 7	95.62% $\pm {0.10}$	96.54% $\pm {0.42}$	93.34% $\pm {0.35}$	93.22% $\pm {0.44}$	96.27% $\pm {0.25}$	96.30% $\pm {0.17}$	94.74% $\pm {0.35}$	96.35% $\pm {0.29}$
3 8	95.49% $\pm {0.51}$	96.43% $\pm {0.65}$	93.35% $\pm {0.48}$	93.22% $\pm {0.52}$	96.05% $\pm {0.32}$	96.09% $\pm {0.61}$	94.44% $\pm {0.33}$	96.14% $\pm {0.66}$
5 3	95.23% $\pm {0.34}$	96.25% $\pm {0.50}$	93.53% $\pm {0.32}$	93.46% $\pm {0.19}$	96.43% $\pm {0.81}$	96.45% $\pm {0.21}$	94.99% $\pm {0.34}$	96.53% $\pm {0.65}$
5 5	95.36% $\pm {0.78}$	96.32% $\pm {0.34}$	93.16% $\pm {0.56}$	93.03% $\pm {0.41}$	96.00% $\pm {0.71}$	96.04% $\pm {0.80}$	94.59% $\pm {0.91}$	96.23% $\pm {0.87}$
5 7	95.62% $\pm {0.40}$	96.53% $\pm {0.61}$	93.25% $\pm {0.21}$	93.14% $\pm {0.53}$	96.21% $\pm {0.10}$	96.25% $\pm {0.23}$	94.94% $\pm {0.31}$	96.48% $\pm {0.40}$
7 3	95.36% $\pm {0.81}$	96.36% $\pm {0.21}$	93.63% $\pm {0.30}$	93.56% $\pm {0.22}$	96.21% $\pm {0.57}$	96.24% $\pm {0.45}$	94.89% $\pm {0.18}$	96.46% $\pm {0.15}$
7 5	95.62% $\pm {0.20}$	96.54% $\pm {0.30}$	93.44% $\pm {0.82}$	93.36% $\pm {0.44}$	95.94% $\pm {0.20}$	95.96% $\pm {0.19}$	94.49% $\pm {0.21}$	96.17% $\pm {0.41}$
7 7	95.76% $\pm {0.29}$	96.64% $\pm {0.30}$	93.44% $\pm {0.29}$	95.94% $\pm {0.91}$	95.97% $\pm {0.30}$	96.00% $\pm {0.10}$	94.59% $\pm {0.90}$	96.24% $\pm {0.12}$
7 8	95.49% $\pm {0.12}$	96.42% $\pm {0.20}$	93.16% $\pm {0.51}$	93.03% $\pm {0.11}$	95.99% $\pm {0.29}$	96.04% $\pm {0.21}$	94.44% $\pm {0.34}$	96.14% $\pm {0.37}$

### Sensitivity evaluation

We have also evaluated the performance sensitivity of GML w.r.t the number of extracted semantic relations and the number of extracted KNN relations respectively. Both parameters are set within the range between 3 and 8. The detailed evaluation results have been presented in Table 6. It can be observed that the performance of GML is very robust w.r.t both parameters. These experimental results bode well for its applicability of GML in real scenarios.

### Discussion on extendability to other classification tasks

It can be observed that our proposed approach leverages binary label relations, which is a general mechanism for knowledge conveyance, to enable gradual learning. For other classification tasks, e.g., aspect-level or document-level sentiment analysis, and even the more general problem of text classification, generating KNN-based relational features is straightforward due to the availability of DNN classifiers. The proposed semantic deep network can also be easily generalized to these tasks, even though technical details need to be further investigated. For instance, for aspect-term sentiment analysis, the input to semantic deep network can be structured as “[CLS] + text1 + [SEP] + aspect1 + [SEP] + text2 + [SEP] + aspect2 + [SEP]”. For document-level sentiment analysis, since the existing pre-trained language models are usually limited to sequences up to 512 characters long, the input to semantic deep network needs to be extended to handle entire documents. Finally, it is noteworthy that the open-sourced GML platform supports the construction of multi-label factor graph and its gradual inference. Therefore, the proposed approach can be potentially extended to handle other binary and even multi-label text classification tasks.

## Conclusion

In this paper, we have presented a novel solution based on GML for the task of sentence-level sentiment analysis. The proposed solution leverages the existing DNN models to extract polarity-aware binary relation features, which are then used to enable effective gradual knowledge conveyance. Our extensive experiments on the benchmark datasets have shown that it achieves the state-of-the-art performance. Our work clearly demonstrates that gradual machine learning, in collaboration with DNN for feature extraction, can perform better than pure deep learning solutions on sentence-level sentiment analysis.

Future work can be pursued in two directions. Firstly, in many practical scenarios, accurately labeled training data may not be readily available. Therefore, it is important to investigate gradual machine learning in the weakly supervised setting, where only a few labeled samples are provided. Secondly, it is interesting to extend the proposed approach to other binary, even multi-label classification tasks.

## Data Availability

All the datasets used in this study are publicly available. MR dataset is available from https://www.cs.cornell.edu/people/pabo/movie-review-data/. CR dataset is available from https://www.cs.uic.edu/~liub/FBS/sentiment-analysis.html#datasets. Twitter2013 dataset is available from https://www.dropbox.com/s/byzr8yoda6bua1b/2017_English_final.zip?file_subpath=%2F2017_English_final%2FGOLD%2FSubtask_A. SST dataset is available from http://nlp.stanford.edu/sentiment. The sentiment lexicon EPA used in our paper is available from http://www.indiana.edu/~socpsy/public_files/EnglishWords_EPAs.xlsx, and another sentiment lexicon VAD is available from https://saifmohammad.com/WebPages/nrc-vad.html.

## Code availibility

Our code is available at: https://github.com/sujingxd/SLSA-GML.
